# Reliability of the TekScan MatScan® system for the measurement of postural stability in older people with rheumatoid arthritis

**DOI:** 10.1186/1757-1146-5-21

**Published:** 2012-08-13

**Authors:** Angela Brenton-Rule, Joshua Mattock, Matthew Carroll, Nicola Dalbeth, Sandra Bassett, Hylton B Menz, Keith Rome

**Affiliations:** 1 AUT University, Health & Rehabilitation Research Institute, Auckland, New Zealand; 2 University of Auckland, Auckland, New Zealand; 3 Musculoskeletal Research Centre, Faculty of Health Sciences, La Trobe University, Bundoora, Victoria, Australia

**Keywords:** Postural sway, Balance, Falls, Rheumatoid arthritis, Pressure system

## Abstract

**Background:**

Postural stability can be measured in clinical and research settings using portable plantar pressure systems. People with rheumatoid arthritis (RA) have decreased postural stability compared to non-RA populations and impaired postural stability is associated with falls in people with RA. The purpose of this study was therefore to investigate the reliability of the TekScan MatScan® system in assessing postural stability in people with RA.

**Methods:**

Twenty three participants with RA, mean (SD) age 69.74 (10.1) years, were assessed in barefoot double-limb quiet standing, with eyes open and eyes closed, for antero-posterior and medio-lateral postural sway values. Three repetitions, at a sampling frequency of 40 Hz, were recorded for each test condition to obtain a mean value. Measurements were repeated one hour later. Intraclass correlation coefficients (ICC) with 95% confidence intervals (CI) were calculated to determine between-session reliability. Measurement error was assessed through the calculation of the standard error of the measurement (SEM) and the smallest real difference (SRD).

**Results:**

The system displayed good to excellent reliability for antero-posterior and medio-lateral sway, with eyes open and closed, as indicated by ICC values ranging from 0.84 to 0.92. Measurement error, as evidenced by the SEM, ranged from 1.27 to 2.35 mm. The degree of change required to exceed the expected trial to trial variability was relatively high, compared to mean values, with SRD ranging from 3.08 to 5.71 mm.

**Conclusions:**

The portability and ease of use of the TekScan MatScan® makes it a useful tool for the measurement of postural stability in clinical and research settings. The TekScan MatScan® system can reliably measure double-limb quiet standing in older people, aged 60 to 80 years, with RA.

## Background

Postural stability can be defined as the maintenance of an upright position in quiet standing or the recovery of balance, associated with voluntary movement [1]. In order to maintain postural stability the body’s global centre-of-mass (COM) must remain inside the body’s base of support; as defined by the outer borders of the feet. This requires active neural control, whereby the central nervous system maintains the COM position in space, resulting in tiny oscillatory movements referred to as postural sway [2]. Postural sway can be measured using portable plantar pressure systems, such as the TekScan MatScan®, which records sway parameters as centre of pressure (COP) excursions in an antero-posterior (AP) and medio-lateral (ML) direction.

Rheumatoid arthritis (RA) is a chronic inflammatory disease characterized by synovial inflammation and progressive articular destruction [3]. Foot deformity is common in RA, with 75% of patients reporting foot involvement within four years of diagnosis, increasing to 90% as the disease progresses [4]. An association between foot deformity and foot function in people with RA has been shown in previous studies [3,5-9]. Functional changes, such as muscle weakness, painful joints, altered gait and decreased postural stability can impair balance and affect everyday activities requiring postural control [10-12].

A high incidence of falls in people with RA has been reported in the literature [13,14]. In a study of 253 people with RA, Armstrong et al. [13] found that 33% reported falls in the previous year, with 52% of these falling more than once. Similarly, Fessel and Nevitt [14] reported that 31% of their sample of 570 RA participants fell once per year and 16% fell twice or more. Postural sway has been found to be increased in RA [15] and associated with falls in people with RA [16]. Rome et al. [15] conducted an exploratory study of 19 RA participants and age matched non-RA controls. AP and ML postural sway was measured for 30 seconds, with eyes open and closed, using a force plate. The results showed that RA participants displayed a significantly larger COP excursion in the AP direction during quiet standing, when compared to the non-RA group, suggesting that postural control mechanisms such as ankle strategies are impeded by the RA process. In a one year prospective study of 84 women with RA, Hayashibara et al. [16] reported that 50% of participants fell and increased postural sway was significantly associated with falls in the study group.

The TekScan MatScan® is commonly used in research and clinical settings and has previously been shown to have moderate to good reliability for the measurement of plantar forces and pressures during barefoot walking in healthy children (ICCs 0.58 to 0.99) [17] and adults (ICCs 0.44 to 0.95) [18]. In both studies, interpretation of the ICCs was in accordance with Portney and Watkins [19] whereby values of > 0.75 indicate good reliability, values ranging from 0.5 to 0.75 imply moderate reliability and values < 0.5 suggest poor reliability.

However the reliability of the TekScan MatScan® for assessing postural sway in double-limb quiet standing has not been evaluated. Previous studies have demonstrated postural stability changes in RA and an association between increased postural sway and falls in an RA population [15,16]. Further investigation into the relationship between postural stability and falls in RA is warranted and there is a need to ensure the equipment used to measure postural sway variables is reliable in this population. Therefore, the primary objective of this study is to determine the between-session reliability of COP based measures of postural control in RA participants using the TekScan MatScan® system.

## Methods

### Participants

Twenty three participants with RA, all meeting the American College of Rheumatology diagnostic criteria [20], were recruited from an outpatient clinic based at AUT University, Auckland, New Zealand. Participants were excluded from the study if they were younger than 18 years, were diagnosed with a neurological condition which could impair balance; such as multiple sclerosis, Parkinson’s disease and history of stroke; lower limb amputation and diabetes with previously diagnosed peripheral neuropathy.

### Clinical characteristics

Clinical characteristics including age, ethnicity, gender, body mass index (BMI), disease duration, co-morbidities, revised Health Assessment Questionnaire (HAQ-II) [21] and pharmacological management, were recorded for each participant. Pharmaceuticals included non-steroidal anti-inflammatory drugs (NSAIDs), Methotrexate, other disease modifying anti-rheumatic drugs (DMARDs), prednisone and biologic therapies.

### Equipment

The TekScan MatScan® pressure mat model 3150 (TekScan Inc, South Boston, USA) was used to capture postural sway values over two sessions. The TekScan MatScan® is a low profile floor mat (5 mm thick) consisting of 2288 resistive sensors (1.4 sensors/cm^2^) with a sampling frequency of 40 Hz. The mat provides measures of AP and ML sway parameters described as; area and direction of sway, distance and direction travelled by the COP and variability of distance travelled by the COP [22]. In the current study, AP and ML sway were measured using the excursion (mm) of the COP in the AP and ML directions. The Sway Analysis Module (SAM™) software was used in conjunction with the TekScan MatScan® to analyze the sway data. One examiner (JM) assessed all the participants. Prior to the commencement of the study, the examiner underwent training in the use of the TekScan MatScan® and interpretation of data using the TekScan SAM™ software.

### Procedure

The AUT University Ethics Committee approved the study. Written informed consent was given by all participants prior to testing. Participants were tested in barefoot double-limb quiet standing on two separate occasions approximately one hour apart. A one hour interval was chosen for practical purposes to enable data collection to occur during the participant’s scheduled podiatry appointment. The one hour interval also ensured that the clinical characteristics of the participants remained consistent. During the period between sessions, participants were provided with a podiatry assessment and treatment as required. To avoid fatigue, the podiatry appointment was conducted in an adjoining room. During testing each participant was directed to step onto the TekScan MatScan® pressure mat and stand in their natural angle and base of gait with their arms by their sides looking straight ahead. To enable the foot position to be replicated from trial to trial, a template was created for each individual according to their preferred barefoot quiet standing position [23]. In order to prevent vestibular disruption and head movement, head position was standardized by asking each participant to focus on the centre of a visual target. The visual target, a 2 cm diameter white spot, was positioned on a screen 2 m in front of the pressure mat at eye level [24]. Participants were asked to remain in this position for a period of 30 seconds while postural sway data was recorded. Participants were tested with eyes open (EO) then eyes closed (EC). Trials were repeated three times for each eye condition to obtain a mean value. Each participant was asked to step backwards off the pressure mat and sit for 30 seconds between repetitions to avoid fatigue. The testing protocol was in accordance with a previous study which used the TekScan MatScan® system to evaluate postural sway in healthy older adults [24].

### Data analysis

Data were analyzed using SPSS V18. Alpha was set at 0.05. All continuous data were screened for normality using the K-S (Kolmogorov-Smirnov) one-sample test. The mean (SD) was obtained for all continuous data. Intraclass Correlation Coefficients (ICC, 2,1) with 95% confidence intervals (CI) were applied to determine between-session reliability of mean sway measurements using a two way mixed effects model with consistency definition [25]. Reliability findings were interpreted by arbitrary benchmarks initially proposed by Fleiss [26]. The strength of the agreement was deemed poor if the correlation ranged from 0 to 0.40; fair to moderate if the correlation ranged from 0.40 to 0.75 and excellent if the correlation ranged from 0.75 to 1.00. Standard error of the measurement (SEM) and SEM% were calculated to assess the difference between the actual measured score and the estimated true scores [27]. The smallest real difference (SRD) was calculated from the SEM to indicate the degree of change that would exceed the expected trial to trial variability [28]. The SEM, SEM% and SRD were calculated as follows: SEM = SD√1-ICC, SEM% = (SEM/mean) x100, SRD = SEM × √2 × 1.717 (where 1.717 represents the *t* value of distribution for a 95% CI (*df* = 22). Bland-Altman plots were calculated to demonstrate graphical representation of key reliability findings. The Bland and Altman method calculates the range within which the difference between the two sessions will lie within a probability of 95% [29]. The use of ICC’s and Bland-Altman plots provide complementary information, as shown by Rankin and Stokes [30].

## Results

All participants completed the trials. No outliers were identified. Participant characteristics are presented in Table 1. All participants were European and most were female. Descriptive statistics for postural sway values are presented in Table 2. The data were normally distributed.

**Table 1:** Demographic and clinical characteristics

**Variable**	**Value**
Age, years, mean (SD), range	69.74 (10.14) 36
Female sex, n (%)	21 (91%)
Ethnicity, n (%)	
European	23 (100%)
Disease duration, years, mean (SD), range	24.24 (12.6) 54
BMI, kg/m^2^, mean (SD), range	26.7 (5.7) 24.1
Revised Health Assessment Questionnaire, mean (SD), range	1.14 (0.56) 1.8
Co-morbidities	
Diabetes, n(%)	3 (13%)
Hypertension	7 (30%)
Other cardiovascular disease, n(%)	4 (17%)
Osteoporosis, n(%)	2 (9%)
Anaemia, n(%)	2 (9%)
Medications	
Methotrexate, n(%)	15 (65%)
Other DMARD, n(%)	8 (35%)
NSAID, n(%)	12 (52%)
Biologics, n(%)	3 (13%)
Corticosteroids, n(%)	9 (39%)

**Table 2:** Descriptive statistics for AP and ML sway (between sessions)

**Sway direction**	**Eye condition**	**Session 1**	**Session 1**	**Session 2**	**Session 2**
		**mean(SD) mm**	**range mm**	**mean(SD) mm**	**range mm**
AP	EO	14.45 (5.44)	5.52-25.20	14.63 (6.10)	6.62-29.97
	EC	18.24 (7.07)	6.81-33.51	18.72 (7.71)	6.73-39.97
ML	EO	8.47 (2.82)	4.71-15.39	9.86 (4.27)	4.60-22.61
	EC	10.62 (4.48)	4.08-24.35	10.29 (4.83)	5.18-22.91

*AP*: antero-posterior; *ML*: medio-lateral; *EO*: eyes open, *EC*: eyes closed.

The relative reliability between sessions, when using the mean measurement for AP and ML sway with eyes open and closed, was good to excellent, as evidenced by ICCs ranging from 0.84 to 0.92 (Table 3). The SEM, SEM% and SRD values consistently showed a moderate level of measurement error, SEM 1.27 to 2.35 mm, SEM% 12.13 to 14.51%, SRD 3.08 to 5.71 mm (Table 3).

**Table 3:** Between-session reliability

**Sway**	**ICC**	**95% CI**	**SEM (mm)**	**SEM%**	**SRD (mm)**
AP sway EO	0.89	0.75-0.96	1.79	12.30	4.35
AP sway EC	0.89	0.74-0.95	2.35	12.70	5.71
ML sway EO	0.84	0.63-0.93	1.33	14.51	3.23
ML sway EC	0.92	0.81-0.97	1.27	12.13	3.08

*AP*: antero-posterior; *ML*: medio-lateral; *EO*: eyes open, *EC*: eyes closed.

**Figure 1:**
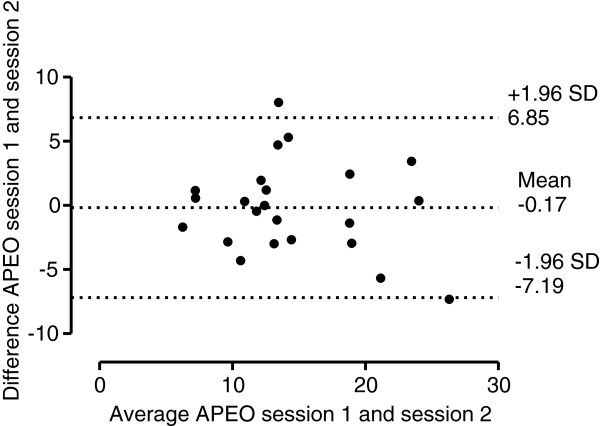
Bland-Altman plot of AP EO measurements (mm).

**Figure 2:**
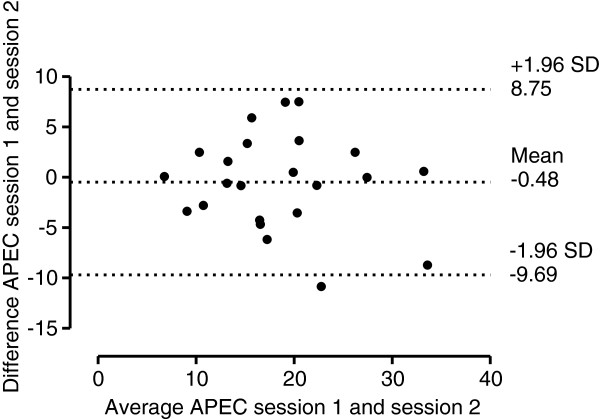
Bland-Altman plot of AP EC measurements (mm).

**Figure 3:**
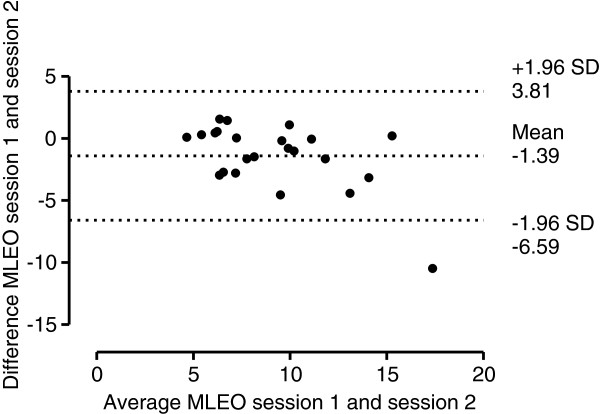
Bland-Altman plot of ML EO measurements (mm).

**Figure 4:**
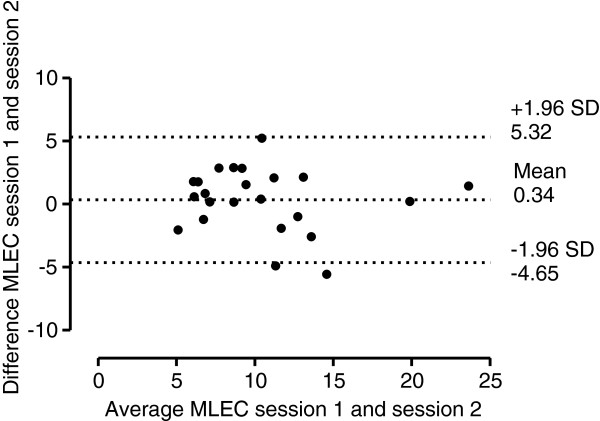
Bland-Altman plot of ML EC measurements (mm).

Figure 1 illustrates the Bland-Altman plot for AP EO measurement in session 1 and 2, with 95% limits of agreement, bias of -0.17 mm (lower limit -7.19 mm, upper limit 6.85 mm). Figure 2 illustrates the Bland-Altman plot for AP EC measurement in session 1 and 2, with 95% limits of agreement, bias of -0.48 mm (lower limit -9.69 mm, upper limit 8.75 mm). Figure 3 illustrates the Bland-Altman plot for ML EO measurement in session 1 and 2, with 95% limits of agreement, bias of -1.39 mm (lower limit -6.59 mm, upper limit 3.81 mm). Figure 4 illustrates the Bland-Altman plot for ML EC measurement in session 1 and 2, with 95% limits of agreement, bias of 0.34 mm (lower limit -4.65 mm, upper limit 5.32 mm).

## Discussion

The reliability of a measurement system used clinically, or in research, must be established in order to be confident in achieving reproducible and meaningful results on different testing occasions. In the current study, the system showed good to excellent between-session reliability in assessing barefoot postural control in double-limb quiet standing, in a sample of older people with RA, as evidenced by ICCs ranging from 0.84 to 0.92. However, measurement error, as expressed by the SEM, SEM% and SRD was relatively high compared to mean values.

The reproducibility of the measures may be attributed to the accuracy of the TekScan MatScan® system in capturing the variables of interest. Indeed the system was found to be highly accurate in an independent study which compared several commonly used plantar pressure measurement systems [31]. Further, due to postural sway values being captured by the measuring system and not the examiner, rater error and bias which may be present in non-computerized tools, such as the sway-meter [32], was minimized.

Measurement error can be due to the precision of the instrument, systematic error introduced by the rater, or the variation in the population being measured [33]. In the current study, measurement error may have occurred as a result of the inherent variability of postural control parameters in the study sample. The wide range in recorded sway values, resulting in a large SD of the mean, supports this possibility. Variation in postural stability parameters within an RA population is to be expected and may be associated with the differing demographic and clinical characteristics of the sample. For example, in a study of 61 patients with RA, Ekdahl [11] found that age, sex and high C-reactive protein level were related to decreased postural control in quiet standing. Given the relatively broad inclusion criteria in the current study it would be expected that this population would display a broad range of demographic and clinical characteristics and therefore a potentially wide range of postural sway values. Therefore, in the current study, the relatively high SEM, SEM% and SRD values may be indicative of the variability of the population tested rather than the reliability of the equipment used to test the population.

It can be further argued that SEM and SRD values are of more relevance in the analysis of within-subject variability. Indeed in a clinical setting measures of postural stability would be undertaken on individuals not populations and the ability to detect a real change in the variables measured over time is essential. As the study aim was to assess between-session reliability over 1 hour, within-subject variability was not analyzed however it is acknowledged that such analysis would be valuable in determining the potential measurement error of the system in a clinical setting.

Postural stability is controlled by the central nervous system. Afferent input from the somatosensory, visual and vestibular systems combine with coordinated muscle activity to maintain balance in quiet standing. In healthy adults, postural control is maintained through flexible and smooth interaction between these systems in order to maintain a stable equilibrium [34]. This may not be the case in an RA population as the ability to maintain balance in quiet standing has been shown to be decreased compared to healthy controls [15]. Variability in postural control between participants was found in the current study as demonstrated by the relatively high degree of measurement error. For this reason, it was necessary to assess the reliability of the TekScan MatScan® system in measuring postural control in an RA group specifically, as this is a population of particular interest to the researchers.

Postural control is a dynamic phenomenon that changes over time. As such, variability in COP values can be expected within individuals and should be accounted for through repetition of test measurements to obtain a mean value [35]. In the current study, the mean of three test measurements of 30 seconds was taken. This was in accordance with a previous study which found that three measurements were sufficient for obtaining a consistent average for dynamic plantar pressure measurements in patients with foot problems associated with chronic arthritis [36]. Due to the variability of COP variables, comparison of individual measurements, i.e. within-session reliability, was not undertaken in the current study.

The role of vision in postural control is well documented and is of particular importance in older adults [34]. Previous studies have demonstrated that removing visual feedback increases postural sway compared to EO test conditions [32,37]. Whilst the current study was interested in adults with RA aged 18 years and older, the cohort age range was 60 to 80 years and hence can be defined as older adult. Our results showed an increase in AP and ML postural sway with the eyes closed condition compared to eyes open condition, which is in agreement with previous studies [24,32,37,38]. Further, when assessing AP and ML sway in an RA population compared with healthy controls, Rome et al. [15] found that, while both groups demonstrated greater sway in eye closed conditions compared to eyes open, the effect was more marked in the RA group. It is important therefore that postural stability in an RA population is assessed with eyes open and eyes closed and hence eyes open and eyes closed test conditions were used to assess the reliability of the equipment in the current study.

The TekScan MatScan® is a portable pressure system commonly used in research and clinical settings to capture and reproduce plantar pressure measures of dynamic foot function. The reliability of the system to accurately and consistently capture dynamic measures has been previously shown [17,18]. The results of the current study suggest that the TekScan MatScan® is reliable for assessing postural control in double-limb quiet standing in older adults with RA. Research implications include the ability to gain a better understanding of the changes in postural stability that occur with age [2] and diseases that affect the feet, such as diabetes and RA. Clinical implications include the ability to identify and manage, through podiatric intervention, patients who are at increased risk of falling. The system may also be useful in evaluating the efficacy of clinical interventions, such as pathological callus debridement, foot orthoses and therapeutic footwear, in reducing postural sway in RA patients.

We acknowledge the limitations of this study. The study cohort was not representative of the general RA population, as all participants were over the age of 60 years. RA affects women three times more than men and peak age at onset is most commonly the fifth decade, although a shift towards older age at onset has been seen in recent studies [39]. A sampling frequency of 40 Hz is relatively low, compared to laboratory based force plate technology, however we believe it is acceptable for measuring the postural sway parameters of interest. Inflammatory disease activity was not assessed as part of the study protocol, and therefore it is not possible to assess the impact of disease activity on variability of the instrument. The study does not address the validity of the TekScan MatScan® in assessing postural control in quiet standing. The validity of a measurement tool can be described as its ability to measure what it is supposed to measure [40]. The validity of the TekScan MatScan® system has been reported by the manufacturer [18] however independent assessment comparing the TekScan MatScan® to force-platform technology would be valuable.

The reliability of the TekScan MatScan® for assessing double-limb quiet standing in healthy adults would be useful. Future investigations should also explore the reliability of the system during more complex dynamic balance tests, in people with RA, as well as other populations of interest such as patients with diabetes or older adults with a history of falls. Testing of the reliability of postural control measures in participants’ usual footwear will also be of interest.

## Conclusion

The portability and ease of use of the TekScan MatScan® makes it a useful tool for use in research and clinical practice. The results of the current study demonstrated good to excellent between-session reliability of postural control measures in older people with RA using the TekScan MatScan® pressure mat.

## Competing interests

ABR, JM, MC, ND, SB and KR have no competing interests to declare. HBM is Editor-in-Chief of the Journal of Foot and Ankle Research. It is journal policy that editors are removed from the peer review and editorial decision-making processes for papers they have authored or co-authored.

## Authors’ contributions

ABR and KR designed the study. JM collected and inputted the data. ABR and MC conducted the statistical analysis. ABR drafted the manuscript with assistance from KR, ND, MC, SB and HBM. All authors approved the final manuscript.
